# Cost-effective interventions to prevent prescription drug misuse: a systematic review

**DOI:** 10.3389/fpubh.2025.1514851

**Published:** 2025-03-04

**Authors:** L. Yesenia Rodríguez-Tanta, Amanda Summers, Fadia T. Shaya

**Affiliations:** Department of Practice, Sciences and Health Outcomes Research, University of Maryland School of Pharmacy, Baltimore, MD, United States

**Keywords:** prescription drug misuse, cost-effectiveness interventions, prescription opioid misuse, cost-utility, opioids

## Abstract

**Background:**

Prescription drug misuse (PDM), which involves the overprescription or inappropriate use of medications such as opioids, benzodiazepines, and stimulants, is one of the primary drivers of the opioid crisis. Identifying and understanding the most cost-effective interventions for preventing PDM is crucial.

**Objectives:**

To conduct a systematic review to identify and synthesize recent cost-effectiveness studies of interventions to prevent PDM.

**Search methods:**

We searched MEDLINE, EMBASE, Scopus, PsycINFO, EconLit, and Tufts CEA Registry from January 2019 until June 2024 to identify cost-effectiveness or cost-utility analyses.

**Selection criteria:**

We included comprehensive economic evaluations addressing our research PICO question.

**Data collection and analysis:**

Two reviewers independently screened and selected studies for inclusion, extracted study information, and assessed the quality of all included studies. The findings were synthesized narratively to provide a comprehensive overview.

**Main results:**

We identified eight recent interventions of fair to good quality that focus on addressing PDM, but none of them addressed benzodiazepines or stimulants. These interventions involved modifications in prescribing behavior, distribution of naloxone in community pharmacies, the use of medication for opioid use disorder with “treatment add-ons,” and education-based strategies. Variations in time horizons, comparison groups, and modeling assumptions led to differences in cost-effectiveness and quality-adjusted life years (QALYs). Nonetheless, all interventions were deemed cost-effective, particularly from a healthcare perspective.

**Conclusion:**

Evidence suggests that while the identified interventions for preventing PDM are cost-effective, their scope remains limited. Further research is needed to address the misuse of other prescription drugs and to evaluate the cost-effectiveness of Prescription Drug Monitoring Programs (PDMPs), particularly their impact on clinicians’ prescribing practices for patients with chronic opioid use. Additionally, incorporating societal perspectives in future studies will be crucial to enhancing policy decisions and developing comprehensive strategies to combat prescription drug misuse globally.

## 1 Introduction

Prescription drug misuse (PDM) involves the overprescribing or inappropriate use of medications like opioids, central nervous system depressants, and stimulants, which can have severe consequences. According to the American Medical Association, between 3 and 19% of patients prescribed pain medications develop an addiction to them, and 45% switch to more accessible narcotics. This increases the risk of chronic opioid use disorder (OUD) and fatal overdoses (1). According to the CDC, between 1999 and 2016, over 350,000 Americans died due to overdoses linked to the use of prescription opioids, contributing to the ongoing opioid crisis (2). Concerning central nervous system depressants, approximately 13% of adult Americans use benzodiazepines, with 2% experiencing a use disorder, which may cause cognitive impairment and a risk of accidents and injuries. In 2019, an estimated 9,720 deaths were attributed to benzodiazepine-related overdoses (3). Prescription stimulant misuse is more prevalent among young adults, with about 12.8% reporting use, 5.85% reporting misuse, and 0.6% developing a stimulant use disorder in 2019. Such misuse is associated with cardiovascular conditions and mental health issues (4).

Moreover, PDM contributes to public health issues and has financial implications such as increased healthcare costs (5), loss of productivity, criminal justice expenses, and it imposes a social burden on families and communities (6, 7). Addressing these issues requires effective, cost-efficient, feasible, and sustainable approaches. Preventive interventions are crucial for addressing PDM and can be classified into primary, secondary, or tertiary prevention. Primary prevention aims to diminish the onset of PDM through education, proper prescribing practices, and regulatory policies. Secondary prevention focuses on early identification and intervention among individuals at risk of PDM. Tertiary prevention targets individuals already affected by PDM to minimize harm.

Cost-effective interventions are crucial for preventing PDM. While there have been numerous reviews of extensive economic evaluations of interventions targeting opioids, cannabis, and illicit drug use, as well as interventions to mitigate drug overuse disorders, they do not specifically focus on PDM (8–11). This study addresses the existing gap by synthesizing recent evidence on interventions aimed at preventing PDM and evaluating their cost-effectiveness. Focusing exclusively on PDM, this systematic review seeks to identify and critically assess the latest cost-effectiveness studies of these preventive interventions. In doing so, it aims to determine which interventions are both the most effective and economically viable, offering valuable insights to guide policymakers and healthcare providers in optimally allocating resources to achieve the best possible outcomes.

## 2 Methods

We performed a systematic review of available articles published in the last 5 years, on the cost-effectiveness of prescription drug misuse prevention. We followed the Cochrane Handbook for Systematic Reviews of Interventions (12). We adhered to the recommendations described in the ISPOR Criteria for Cost (−Effectiveness) Review Outcomes (CiCERO) Checklist (13) for the economic aspects. We also followed the guidelines of the Preferred Reporting Items for Systematic Reviews and Meta-Analyses (PRISMA) statement for reporting the process (14).

### 2.1 Search strategy

We created a search strategy from January 2019 to June 2024 using the following databases: (1) MEDLINE (as of 10th June 2024), (2) EMBASE (as of 10th June 2024), (3) Scopus, (4) PsycINFO, (5) EconLit, and (6) Tufts CEA Registry. We tailored the search algorithms to the specifications of each electronic database and employed validated filters to obtain suitable designs as required. Additionally, we examined the reference lists of previous systematic reviews for potentially eligible studies.

### 2.2 Eligibility criteria

We included comprehensive economic evaluations addressing our research question (see structured PICO question). These evaluations conducted comparative analyses of interventions, examining costs and consequences (outcomes and effects) through cost-effectiveness and cost-utility analysis. Our review encompassed model-based studies using a lifetime horizon and empirical health economic studies using shorter horizons, including economic evaluations based on randomized and non-randomized trials. We excluded cost–benefit studies, partial economic evaluations, conference abstracts, letters to the editor, and studies not published in English.

− Population: Individuals of any age who are prescribed opioids for chronic or non-chronic pain or post-surgical recovery, as well as those who are prescribed depressants or stimulants for anxiety disorders. This also includes individuals with a history of substance abuse who have received prescriptions for any of these medications.
− Intervention: Primary and/or secondary prevention aimed at preventing PDM.
− Comparator: No intervention or standard of care (usual care).
− Outcomes: incremental cost-effectiveness ratio (ICER) expressed by either quality-adjusted years (QALYs) or Life-years (LY) gained, PMD-specific outcomes (i.e., risk of drug abuse, overdose rates avoided (fatal and nonfatal), time of substance abstinence, reduction in hospitalizations, crime rates avoided, etc.).

### 2.3 Selection process and data extraction

Two reviewers (LYR, AS) initially assessed search results based on titles and abstracts, followed by full-text reading. Disagreements were resolved by consulting a third reviewer. One reviewer (LYR) extracted the main characteristics of included studies in a pre-designed form, including (1) general information (authors, publication year, country, conflicts of interests); (2) study characteristics (type of intervention, substance, comparator); (3) methodology (model, type of economic evaluation, perspective, time horizon, discounting rate, currency, sensitivity analysis, sources of information); and (4) cost-effectiveness outcomes. A second reviewer performed a quality control of the extraction process (AS).

### 2.4 Assessment of methodological quality

We assessed the quality of the primary studies that were included by using the Consolidated Health Economic Evaluation Reporting Standards (CHEERS) 2022 checklist developed by ISPOR (15). The checklist consists of 28 items grouped into six categories: (1) title and abstract, (2) introduction, (3) methods, (4) results, (5) discussion, and (6) others. Each item received one point if it met the quality criteria and zero points if it did not, resulting in a maximum score of 28 for each study. The results were categorized as “Excellent” if all items were present in the study, “Good” if at least 80% were satisfied, “Fair” if at least 70% of the items were satisfied, and “Average” if at least 60% were satisfied. Two researchers (AS, YR) independently assessed the quality of each study. The results were reviewed by FS. Any discrepancies were resolved through discussion and consensus and final decisions with a third researcher (FS).

## 3 Results

### 3.1 Study selection

**Figure 1:**
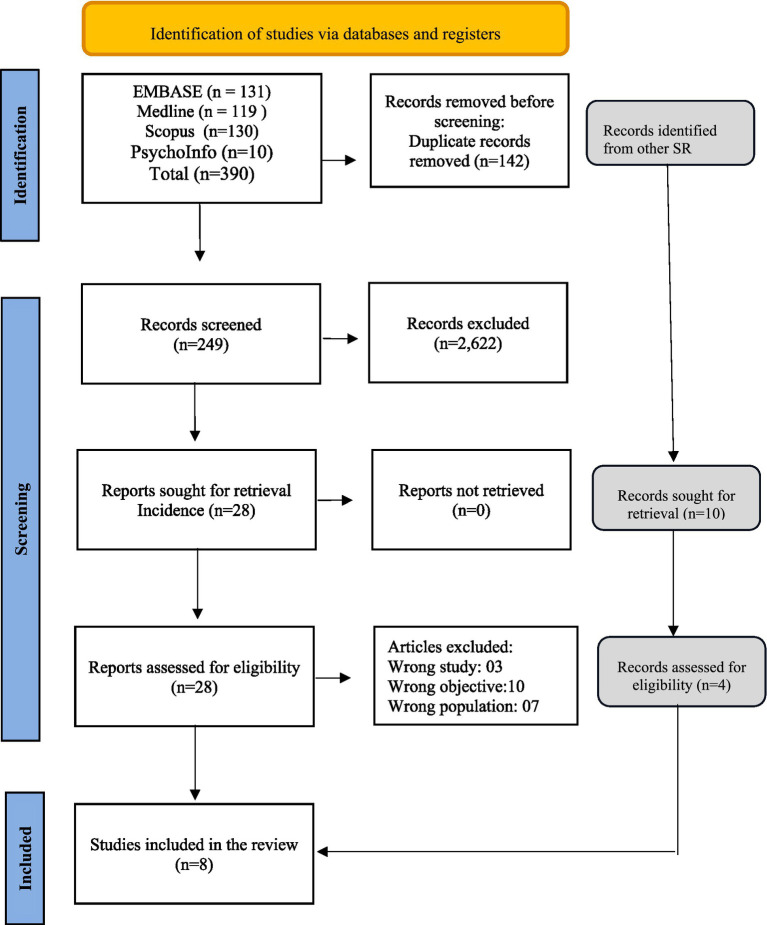
PRISMA flow diagram.

We identified 390 individual records through our search process. After removing duplicates, we screened 248 records based on their titles and abstracts. Subsequently, we evaluated 28 studies in full text and ultimately included eight studies in our review (16–23). Figure 1 depicts the PRISMA flow chart, visually representing our screening process. Supplementary Table S1 provides detailed reasons for excluding specific studies.

### 3.2 Characteristics of the included studies

Table 1 summarizes the characteristics of the included studies. Five studies were conducted in the USA, two in Korea, and one in Canada. Two studies focused on specific contexts in Connecticut and Massachusetts (19, 22). All included interventions concentrated solely on opioids, with none addressing benzodiazepines or stimulants. Costs were expressed in the respective country’s currency. Most studies utilized simulated cohorts constructed with data from relevant sources alongside national and local population estimates. One study was a cost-effectiveness analysis based on a randomized controlled trial (RCT) (22), while three were retrospective analyses using a National Registry (20, 23) and US Army soldiers’ data (21). The articles involved a diverse group of 439 to 2.5 million adults, comprising both men and women exposed to opioids. The only exception was the RCT, in which only women participated. The authors of the five studies declared no conflicts of interest, and funding sources were mainly from government or not-for-profit organizations.

**Table 1:** Characteristics of the studies included in the systematic review.

Study ID	Country (State)	Setting	Population	Drugs description	Group of interventions	Interventions description		Comparator
Acharya 2020 (16)	USA	Retail pharmacies	Microsimulation of 100,000 individuals with a mean age of 48 (SD 12) years.	High-risk prescription opioid (RxO) users.	Expanding access to naloxone (NLX)	pharmacy-based intranasal (IN) NLX distribution (one-time vs. biannual)	(a) One-time (b) biannual follow-up distribution	Standard of care
Cid 2024 (17)	Canada	Community pharmacies	Individuals with an average age of 38 years, equally distributed by sex	Individuals with opioid prescription and illicit opioids, as well as opioid-agonist therapy		pharmacy-based IN and intramuscular NLX distribution	(a) IM NLX distributed by pharmacy (b) IN NLX distributed by pharmacy	No NLX distribution
Claypool 2023 (18)	USA	Mixed	NS	individuals with opioids/ heroin prescription	Interventions involving medications for opioid use disorder (MOUD)	Buprenorphine (BUP) treatment interventions	individually and in combination (32 int) (a) BUP initiation in ED (ED) (b) contingency management (CM), (c) psychotherapy (P), (d) telehealth (TH), (e) hub-and-spoke treatment programs (HS)	Status Quo of BUP prescription
Savinkina 2022 (19)	USA (Massachusetts)	Detox centers	Microsimulation of 40,000	Individuals with OUD who have been prescribed opioids or who use injected opioids		MOUD initiation in DETOX centers	MOUD during DETOX process	Standard of care
Kim 2021 (20)	South Korea	Healthcare setting	NS	individuals without cancer who had been prescribed > = 1 outpatient opioid	Modifications on prescription behavior	Prevention program “Network System to Prevent Doctor-Shopping for Narcotics”	By accessing the system, doctors can examine previous narcotics prescriptions.	No implementation of the program
Kim 2023 (23)			NS	non-cancer patients with chronic opioid use				
Bjarnadóttir 2020 (21)	USA	Primary care centers	827,265 Active-duty US Army soldiers from 2011 to 2014 with an average of 29.14 years (15.25% females)	individuals with initial opioid prescriptions		Reducing initial opioid prescription length.	Three days or shorter	Longer prescription
Olmstead 2019 (22)	USA (Connecticut)	Reproductive Health Centers	439 women visiting two urban, academic reproductive health clinics	Women who used cigarettes, alcohol, illicit drugs, and misused prescription medication	Education-based intervention	SBIRT (Screening, Brief Intervention, and Referral to Treatment)	(a)electronic-delivered SBIRT (e-SBIRT) (b) clinician-delivered SBIRT	Standard of care

IN, intranasal; IM, intramuscular; NLX, Naloxone; BUP: Buprenorphine; MET, Methadone; CM, contingency management; HS, hub-and-spoke treatment programs; ED, BUP initiation in emergency department; TH, telehealth; MAT, Medication-assisted treatment; MOUD, medications for opioid use disorder; OEND, overdose education and naloxone distribution; PT, psychotherapy; NS, Not shown.

Table 2 presents the characteristics of the economic evaluations and their outcomes. Two studies used Markov-based models, two used Markov and decision-tree models, and one used a discrete event simulation (DSE). Two studies used specific models to simulate populations, including RESPOND and SOURCE. Regarding the economic evaluation (EE), five studies were cost-utility analyses, two conducted cost-effectiveness analyses, and one performed a budget impact analysis (19). We categorized EE reporting cost-effectiveness results using quality-adjusted life-years (QALYs) as “cost-utility” analyses. Seven studies conducted sensitivity analyses, with six performing deterministic and probabilistic analyses and one conducting probabilistic analysis only. The most common discount rate used was 3%. The time horizon varied across interventions, ranging from 6 months to a lifetime. Regarding the perspective of the EE, eight studies reported from a healthcare perspective, one adopted a societal perspective, and one considered third-party payers.

**Table 2:** Summary of findings.

Study ID	Model/ Economic evaluation	Time Horizon	Year and currency	Perspective	Sensitivity analysis	Effectiveness outcomes estimation	Effectiveness outcomes results	Cost outcomes estimation	Cost outcomes results	Conclusion
Acharya 2020 (16)	Markov model + DT CEA	Lifetime horizon	2018 US$.	US healthcare	DSA and PSA	Opioid overdose deaths prevented per 100,000 people	14 (one-time); 107 (biannual)	QALYs (SF-12 MCS-12)	0.00173 (one-time), 0.00446 (biannual).	Both one-time and biannual follow-up NLX distribution in community pharmacies would be cost-effective at a WTP of $100,000/ QALY.
								ICER	$56,699/QALY (one-time), $84,799/QALY (biannual).	
Bjarnadóttir 2020 (21)	Markov model	2-year horizon	2011–2014 US$.	US Military Healthcare	DSA and PSA	Additional opioid-free months	4,451	ICER	11,850.84 (constant utility)	Reducing the duration of the initial opioid prescription is cost effective across a wide range of program costs.
								Cost savings	$3.1 million over two years (750 US$ program cost per patient)	
Cid 2024 (17)	Markov model + DT	Lifetime horizon	2020 CAD	Canadian provincial Ministry of Health	DSA and PSA	Opioid overdose deaths prevented per 10,000 people	151	QALYs gained	0.03 (IM NLX); 0.03 (IN NLX)	Distribution of IM and IN naloxone to Canadians every 3 years may be cost-effective at a WTP of $140,000 Canadian dollars/QALY
								ICER	$44,944/QALY (IM NLX) $104,051/QALY (IN NLX)	
Claypool 2023 (18)	SOURCE	12-year time horizon from 2021 to 2032	2021 US$.	Society and healthcare	PSA	Opioid overdose deaths prevented per 10,000 people	3,530 (CM); 2,420 (TH); 1,110 (ED); 940 (HS); 80 (P); 8,570 (CM + HS + ED + TH)	QALYs gained	182,127 (CM) 520,557 (CM + HS + ED + TH + P)	The combination of strategies (CM, hub-and-spoke clinician training, ED BUP initiation, and telehealth) was preferred a at a generally accepted threshold and was likely to be cost-saving compared with the status quo
						Opioid non-fatal overdose prevented per 10,000 p-y	30,400 (CM); 20,900(TH); 10,000 (ED); 9,000 (HS); 700 (P); 75,900 (CM + HS + ED + TH)	ICER	$19,381/QALY (CM + HS + ED + TH)	
Kim 2021 (20)	Markov model	30 years	2019 US$.	Healthcare payer	DSA and PSA	Opioid non-fatal overdose prevented per 100,000 people-years	2.27 person-years	QALYs gained (SF-6 MCS-12)	0.00505	The “Network System to Prevent Doctor-Shopping for Narcotics” is a cost-effective strategy at the WTP threshold of GDP per capita in South Korea ($31362.80 per QALY).
								ICUR	$227/ QALY	
Kim 2023 (23)	DES	30 years	2019 US$.		DSA and PSA	NS	NS	QALYs	0.05 at an additional cost ($110)	Considering patient-level characteristics and abuse history, PDMP based on NIMS was found to be a cost-effective strategy for preventing opioid abuse in South Korea.
								ICUR	$2,227/QALY	
Olmstead 2019 (22)	NS	6-month follow-up	2016 US$.	Healthcare and patient	NS	Number of days of primary substance abstinence	16.66 days (e-SBIRT); 16.48 days (SBIRT)	NS	NS	e-SBIRT may be a cost-effective approach, from both healthcare provider and patient perspectives.
Savinkina 2022 (19)	RESPOND	10-year time horizon (2021 to 2030)	2019 US$.	Healthcare payer	DSA and PSA	Opioid overdose deaths prevented	4.5% (perfect linkage) 2.3% (moderate linkage)	QALYs gained (gamble method)	5,512 (perfect linkage) 2,869 (moderate linkage)	Initiation of medications for OUD and linkage policies among detox patients could prevent fatal opioid overdoses in the OUD population and would be cost-effective from a healthcare sector perspective.
								ICER	$55,600/QALY (perfect linkage) $78,500/QALY (moderate linkage)	

DT, Decision tree; CEA, Cost-effectiveness analysis; DSA, Deterministic sensitivity analysis; PSA, Probability analysis; ICER, Incremental cost-effectiveness ratio; QALYs, Quality-adjusted life year; REDUCE, Reducing infections related to drug use cost-effectiveness model; LYG, Life-years gained; IN, intranasal; IM, intramuscular; NLX, Naloxone; BUP, Buprenorphine; MET, Methadone; CM, contingency management; HS, hub-and-spoke treatment programs; ED, BUP initiation in emergency department; TH, telehealth; MAT, Medication-assisted treatment; MOUD, medications for opioid use disorder; OEND, overdose education and naloxone distribution; PT, psychotherapy; LAP, lay people; PF, police and fire; EMS, emergency medical services; SBIRT, Screening, Brief Intervention, and Referral to Treatment; MOUD-COM, MOUD in the community; MOUD-INC, MOUD in during incarceration; ICUR, Incremental cost-utility ratio; PDMP, prescription drug monitoring program; NIMS, Narcotics Information Management System; NS, Not shown.

In terms of outcomes, all studies reported incremental cost-effectiveness ratios (ICER). Five studies reported quality-adjusted life years (QALYs), and one showed cost savings. Additionally, four studies reported prevention of opioid overdose deaths, two showed preventions of non-fatal opioid overdose, and two reported the number of days/months of drug abstinence. Only one study reported utilizing the CHEERS reporting guidelines.

### 3.3 Interventions to prevent prescription opioid misuse

The interventions targeted diverse populations in various settings, including community pharmacies (16, 17), primary care centers (21), reproductive centers (22), detox centers (19), and other healthcare settings (20). One intervention involved mixed settings, such as emergency departments (ED) and community centers (18). Most interventions focused on ambulatory individuals with opioid use disorder (OUD) who were prescribed opioids. One intervention specifically targeted high-risk prescription opioid users (16). Five interventions were categorized as secondary prevention (16, 18–20, 23), two as primary prevention (21, 22), and one mixed prevention (involving aspects of both primary and secondary preventions) (17). To facilitate the description of the included interventions, we grouped them as follows:

− Modifications on prescribing behavior.
− Expanding access to naloxone (NLX).
− Interventions involving medications for opioid use disorder (MOUD).
− Education-based intervention (Screening, Brief Intervention, and Referral to Treatment (SBIRT)).

### 3.4 Modifications on prescribing behaviors

We identified three studies examining the effectiveness and economic impact of modifying opioid prescribing behaviors (20, 21, 23). A retrospective cohort used a Markov decision process model to evaluate a policy intervention aimed at reducing the length of an initial opioid prescription to 3 days in a military population. This intervention resulted in $3.1 million in cost savings over 2 years and produced around 4,500 additional opioid-free months (21). One South Korean study found that “The Network System to Prevent Doctor-Shopping for Narcotics” program was considered cost-effective over 30 years, with a cost of US$227 per QALY and a WTP threshold of US$31,362 per QALY from a healthcare system perspective. The program was determined to be 100% cost-effective, even with a WTP threshold of US$900 per QALY (20). Another South Korean study that assessed the same intervention using DES found that this strategy was cost-effective, with an estimated ICUR of $2,227/QALY (23).

### 3.5 Interventions to expand access to NLX

These interventions focus on strategies to scale up or expand the distribution of NLX. In this review, two studies evaluated the cost-effectiveness of scaling up NLX distribution (11, 12). The interventions were (a) pharmacy-based distribution of intranasal (IN) NLX (one-time and biannual) in the U.S., and (b) pharmacy-based distribution of both IN and intramuscular (IM) NLX in Canada. The one-time and biannual intranasal NLX distribution strategies were cost-effective, with ICERs of $56,699 per QALY gained and $84,799 per QALY gained, respectively. These values are below the accepted willingness-to-pay (WTP) threshold of $100,000 per QALY gained. Additionally, the one-time distribution prevented 14 additional overdose deaths, while the biannual distribution prevented 107 overdose deaths per 100,000 people.

### 3.6 Interventions involving MOUD

We identified two interventions focused on expanding MOUD treatment in the U.S. (18, 19) MOUD includes buprenorphine (BUP), methadone, or injectable extended-release naltrexone. The interventions included (a) Initiating MOUD during the detox process and ensuring linkage to outpatient care and (b) MOUD + “treatment add-ons.”

The MOUD initiation in detox centers with perfect linkage turned out to be cost-effective compared with the standard of care, with an ICER of $55,600 per QALY, reducing opioid overdose deaths by 4.5% (19). Additionally, one study explored the use of any MOUD in combination with potential “treatment add-ons,” which refer to additional therapies or supports provided in conjunction with a primary treatment to enhance its effectiveness (18). Increasing the capacity and duration of MOUD, particularly BUP, coupled with the provision of additional therapies such as psychotherapy, contingency management, or telehealth, led to an increase in QALYs gained and effectively prevented 8,570 opioids fatal overdoses and 75,900 non-fatal overdoses per 10,000 person-year.

### 3.7 Education-based intervention

A cost-effectiveness analysis was conducted using a randomized controlled clinical trial comparing electronic (e) and clinician-delivered SBIRT (Screening, Brief Intervention, and Referral to Treatment). This intervention aims to reduce primary substance use among women receiving treatment in reproductive health centers in New Haven, CT (United States). The results suggest that e-SBIRT could be a cost-effective approach from both healthcare provider and patient perspectives, increasing the days of abstinence during the 6-month follow-up period by 16.66.

### 3.8 Quality assessment

Table 3 presents the quality assessment characteristics using the 2022 CHEERS checklist. None of the studies were rated as excellent; four were judged to be of good quality, and four were rated as fair quality.

**Table 3:** Quality assessment of the included studies using Consolidated Health Economic Evaluation Reporting Standards 2022 (CHEERS 2022).

Study ID	Title (1 pts)	Abstract (1 pts)	Introduction (1 pts)	Methods (18 pts)	Results (4 pts)	Discussion (1pts)	Other information (2 pts)	Total (28 pts)	Percentage	Quality
Acharya 2020 (16)	1	1	1	16	3	1	0	23	82	Good
Bjarnadóttir 2020 (21)	0	1	1	17	2	1	0	22	79	Fair
Cid 2024 (17)	1	1	1	14	2	1	2	22	79	Fair
Claypool 2023 (18)	1	1	1	15	2	1	2	23	82	Good
Kim 2021 (20)	1	1	1	15	2	1	2	23	82	Good
Kim 2023 (23)	1	1	1	15	2	1	2	22	81	Good
Olmstead 2019 (22)	1	1	1	14	3	1	1	22	79	Fair
Savinkina 2022 (19)	1	1	1	15	2	1	1	22	79	Fair

## 4 Discussion

Our systematic review aimed to identify, synthesize, and critically evaluate cost-effectiveness studies on strategies to prevent Prescription Drug Misuse (PDM) in adults. We identified eight studies of fair to good quality, published within the last 5 years, that focused on both demand- and supply-side interventions to reduce opioid misuse. These interventions included modifications in prescribing behavior, naloxone distribution in community pharmacies, the use of MOUD in combination with potential “treatment add-ons,” and education-based strategies. Most were implemented in the United States across diverse healthcare settings, underscoring the necessity for comprehensive, integrated strategies that target multiple aspects of prescription drug misuse. These findings highlight the complex and multifactorial nature of PDM and the need for multifaceted public health interventions to address it effectively. Given that PDM is a critical driver of the ongoing opioid crisis (26–28), addressing these factors through integrated approaches is essential for mitigating the broader epidemic.

All interventions included in this review demonstrated cost-effectiveness, with ICERs falling well below commonly accepted WTP thresholds from a healthcare perspective. This perspective primarily accounts for direct healthcare costs and outcomes, such as medical expenses and treatment-related benefits. These findings underscore that these interventions not only provide substantial public health benefits in preventing PDM, but they do so at a cost considered economically justifiable within the healthcare system. The fact that ICERs are below the accepted WTP thresholds indicates that these strategies offer excellent value for money, making them strong candidates for inclusion in healthcare funding and policy decisions. However, it is important to note that the healthcare perspective may not fully capture the broader societal impacts of these interventions. Future assessments from a societal perspective could offer a more comprehensive evaluation of the overall value of the interventions, considering their impact on productivity, quality of life, and societal well-being.

We identified three studies that assessed the impact of prescription drug monitoring programs (PDMPs) and changes in clinicians’ prescribing behaviors on the opioid supply. Two of these studies evaluated the cost-effectiveness of an early-stage PDMP in South Korea, known as the “Network System to Prevent Doctor-Shopping for Narcotics,” and concluded that this strategy is cost-effective. However, the broader literature on PDMP effectiveness in reducing opioid misuse is mixed (26, 29, 30), with some studies showing decreased abuse (31–33) and others noting unintended consequences (34). No economic evaluations from other countries limit the applicability of these findings. Additionally, we identified a study evaluating the cost-effectiveness of a policy aimed at limiting initial opioid prescriptions to 3 days for military personnel, in line with CDC guidelines. This intervention was deemed cost-effective over a two-year period, although its effectiveness in chronic conditions was not assessed. This finding reinforces the conclusion of other studies, which highlight that prescribing behaviors remain a critical factor, with physician opioid prescriptions consistently identified as a primary source of initial opioid supply (21, 35, 36).

We also identified interventions aimed at reducing the demand for opioids to prevent prescription drug misuse, resulting in favorable cost and health outcomes. Expanding access to NLX through the simulated implementation of pharmacy-based NLX distribution led to substantial reductions in opioid overdose deaths, as other studies have demonstrated (24, 25). Similarly, when combined with additional treatment enhancements, MOUD interventions, the gold standard for OUD treatment (26), have shown a positive impact, particularly when initiated early in specific settings like detox or primary care centers. MOUD combined interventions reduced the number of fatal and non-fatal opioid overdoses, increased QALYs, and met cost-effectiveness thresholds for clinical adoption and policy. However, it is important to note that these results were not specific to prescription opioid users, as the studies included patients who injected illicit opioids. Furthermore, given that the studies were conducted in the United States, the generalizability of these findings to diverse global contexts may be limited.

While all these preventive interventions have demonstrated positive outcomes, it is important to note that most studies relied on modeling techniques to build cohorts and forecast the long-term impacts and economic outcomes. Therefore, transparency regarding the uncertainty inherent in these projections is essential. The studies included in our review that used modeling techniques typically conducted Monte Carlo simulations and probabilistic sensitivity analyses to address uncertainties and variability in their findings. These methods allowed for the incorporation of a range of plausible parameter values and outcomes, enhancing the robustness of the conclusions (37). However, despite these efforts to account for uncertainty, modeling studies are still subject to limitations such as assumptions about the accuracy of input data, the representativeness of the modeled population, and the external validity of the model to real-world settings. As such, while the results provide valuable insights into the potential cost-effectiveness of interventions, further empirical studies in diverse, real-world contexts are necessary to validate these findings and better inform policy decisions.

Our review aimed to explore preventive interventions for prescription drugs prone to misuse. However, the identified studies focused exclusively on opioids, limiting the generalizability of our findings to other classes of prescription medications such as benzodiazepines, stimulants, or sedatives. This highlights a significant gap in the current literature and underscores the need for future research to comprehensively address interventions targeting a broader range of prescription drugs that are also prone to misuse. Expanding the scope of such research would provide a more holistic understanding of effective strategies and policies for reducing the misuse of various prescription medications, ultimately guiding public health efforts to combat the growing problem of substance misuse across different drug categories.

### 4.1 Strengthens and limitations

This systematic review distinguishes itself from others (8–11) by focusing exclusively on strategies to prevent PDM, particularly on articles published within the last 5 years. This contemporary focus not only highlights the review’s relevance in addressing the cost-effectiveness of the latest interventions on PDM but also provides valuable insights into the ongoing challenges and gaps. Likewise, for reporting results, we adhered to the recommendations described in the ISPOR Criteria for Cost (−Effectiveness) Review Outcomes (CiCERO) Checklist, ensuring a comprehensive and transparent presentation of the economic aspects of our review. As part of our review, we assessed the study quality using the 2022 checklist “Consolidated Health Economic Evaluation Reporting Standards (CHEERS)” developed by ISPOR.

Our review has some limitations. We restricted our search to scientific publications and did not include gray literature or reports from health technology agencies. As a result, our findings may not cover all available evidence, and we cannot definitively claim that these are the only relevant results. In addition, we cannot conclude that the results of the included interventions apply solely to patients who were prescribed opioids, as some of the studies, especially those related to the use of MOUD, included populations exposed to illicit drugs as well. We faced difficulties classifying interventions into three levels of prevention due to incomplete descriptions of the population characteristics. It is crucial to accurately understand the history and the risk of opioid use disorder among participants receiving drug prescriptions or within simulated populations in order to categorize an intervention as secondary or tertiary prevention properly. Without this context, it is challenging to determine the precise level of prevention.

Finally, we excluded studies published in languages other than English, which may have limited the scope and comprehensiveness of our search. By not including research published in other languages, we may have overlooked important studies that could have provided valuable insights, particularly from regions where English is not the primary language. This exclusion may have affected the generalizability of our findings.

### 4.2 Directions for future research

Despite their limitations, the findings of this review hold substantial implications for healthcare policy, practice, and future research directions. There is a critical need for research into the economic evaluations of PDMPs, the impact of clinician adherence to CDC guidelines on PDM prevention, especially in chronic conditions, and the cost-effectiveness of strategies to mitigate or prevent the misuse of prescription benzodiazepines and stimulants. Moreover, advancing economic models, conducting rigorous cost–benefit analyses, and evaluating real-world implementation strategies are critical to ensure evidence effectively informs policy decisions, not only from a healthcare perspective but also from societal and payer perspectives.

## 5 Conclusion

This systematic review identified studies evaluating the cost-effectiveness of interventions to prevent PDM. The interventions, including prescribing behavior modifications, naloxone distribution, medication for opioid use disorder (MOUD) with enhancements, and educational initiatives, demonstrated strong value for money, with ICERs well below commonly accepted thresholds for healthcare expenditure, despite variations in time horizons and comparison groups. While these findings highlight the potential for these interventions to significantly reduce opioid misuse and associated harms, the generalizability of the results is limited by the focus on opioid misuse and the reliance on modeling techniques. Future research should expand to include broader drug classes, such as benzodiazepines and stimulants, assess real-world implementation, and consider societal perspectives to further inform policy decisions and ensure comprehensive, effective strategies to combat PDM globally.

## Data Availability

The original contributions presented in the study are included in the article/Supplementary material, further inquiries can be directed to the corresponding author.
