# Supplementary material for: Cost-effective interventions to prevent prescription drug misuse: a systematic review
Source: Front Public Health. 2025 Mar 4;13:1514851. doi: 10.3389/fpubh.2025.1514851 (PMC11917492; doi:10.3389/fpubh.2025.1514851)
Supplement: Supplementary file 1 [file Supplementary_file_1.pdf]

PMD SEARCH STRATEGY

EMBASE (n=131)

('opioid' OR 'narcotic' OR 'morphine' OR 'oxycodone' OR 'hydrocodone' OR 'fentanyl' OR 'codeine' OR 'benzodiazepine' OR 'diazepam' OR 'lorazepam' OR 'alprazolam' OR 'stimulant' OR 'amphetamine' OR 'methylphenidate' OR 'Adderall' OR 'Ritalin' OR 'sedative' OR 'tranquilizer' OR 'barbiturate' OR 'sleep aid' OR 'hypnotic') AND ('misuse' OR 'abuse' OR 'inappropriate use' OR 'non-medical use' OR 'overuse' OR 'overprescribing' OR 'dependence' OR 'addiction' OR 'substance use disorder' OR 'overdose' OR 'poisoning') AND ('cost-effectiveness' OR 'cost benefit' OR 'cost utility' OR 'economic evaluation' OR 'cost analysis' OR 'health economics' OR 'budget impact') AND ('prevention' OR 'mitigation' OR 'prevention strategies' OR 'preventive measures' OR 'harm reduction' OR 'risk reduction' OR 'early intervention')

PUBMED (n=119)

(("opioid*" OR "narcotic*" OR "morphine" OR "oxycodone" OR "hydrocodone" OR "fentanyl" OR "codeine") OR ("benzodiazepine*" OR "diazepam" OR "lorazepam" OR "alprazolam") OR ("stimulant*" OR "amphetamine" OR "methylphenidate" OR "Adderall" OR "Ritalin") OR ("sedative*" OR "tranquilizer*" OR "barbiturate*" OR "sleep aid*" OR "hypnotic*"))AND ("misuse" OR "abuse" OR "inappropriate use" OR "non-medical use" OR "overuse" OR "overprescribing" OR "dependence" OR "addiction" OR "substance use disorder" OR "overdose" OR "poisoning") AND ("cost-effectiveness" OR "cost benefit" OR "cost utility" OR "economic evaluation" OR "cost analysis" OR "health economics" OR "budget impact") AND ("prevention" OR "mitigation" OR "prevention strategies" OR "preventive measures" OR "harm reduction" OR "risk reduction" OR "early intervention"))

SCOPUS (n=130)

(TITLE-ABS-KEY ("opioid misuse" OR "narcotic misuse" OR "morphine misuse" OR "oxycodone misuse" OR "hydrocodone misuse" OR "fentanyl misuse" OR "codeine misuse") OR TITLE-ABS-KEY ("benzodiazepine misuse" OR "diazepam misuse" OR "lorazepam misuse" OR "alprazolam misuse") OR TITLE-ABS-KEY ("stimulant misuse" OR "amphetamine misuse" OR "methylphenidate misuse" OR "Adderall misuse" OR "Ritalin misuse") OR TITLE-ABS-KEY ("sedative misuse" OR "tranquilizer misuse" OR "barbiturate misuse" OR "sleep aid misuse" OR "hypnotic misuse")) AND ("cost-effectiveness" OR "cost-benefit" OR "cost-utility" OR "economic evaluation" OR "cost analysis" OR "health economics" OR "budget impact") AND ("prevention" OR "mitigation" OR "prevention strategies" OR "preventive measures" OR "harm reduction" OR "risk reduction" OR "early intervention") AND PUBYEAR > 2018 AND PUBYEAR < 2025

PsycInfo (n=8) 2019-2024

('cost-effectiveness' OR 'economic evaluation' OR 'cost-benefit analysis') AND ('substance abuse' OR 'substance misuse' OR 'drug abuse' OR 'drug misuse') AND ('opioids' OR 'prescription opioids' OR 'benzodiazepines') AND ('prevention' OR 'intervention' OR 'preventive measures' OR 'harm reduction') AND ('effectiveness' OR 'efficacy' OR 'impact' OR 'outcome') AND ('cost-effective' OR 'cost utility' OR 'cost analysis' OR 'cost saving' OR 'cost efficiency') AND ('review' OR 'evaluation' OR 'study' OR 'trial' OR 'assessment')

Table S1 List of excluded studies and the main reason for exclusion

Study ID	Main reason for studying	
Al Zahrani 2020	Wrong study	It is not a cost-effectiveness analysis (CEA)
Behrends 2019	Wrong objective	It aims to evaluate the potential impact on opioid overdose fatalities and healthcare system costs of implementing SIFs. No CEA, no comparator
Cepeda 2023	Wrong objective	This study is a cost analysis of a new pilot drug checking, which is not the objective of our review.
Flam-Ross 2023	Wrong objective	Tertiary prevention (treatment in clinical treatment)
Hood 2019	Wrong objective	It aims to evaluate the potential impact on opioid overdose fatalities and healthcare system costs of implementing SIFs. No CEA, no comparator
Idrisov 2017	Wrong objective	Tertiary prevention (clinical treatment). It lacks clarity regarding the target population's prescription drug misuse and is based on data older than 2019.
Irwin 2017	Wrong study	It is a cost-benefit analysis
Kumar 2019	Wrong objective	Our review included patients experiencing prescription drug overuse. This study is contemplating a shift to a deterrent formulation.
Morozova 2020	Wrong objective	The study only mentions patients with overuse disorder, but it is not stated whether this is due to prescription drug misuse.
Murphy 2019	Wrong objective	Tertiary prevention (treatment in clinical treatment)
Naumann 2019	Wrong objective	The study only mentions patients with overuse disorder, but it is not stated whether this is due to prescription drug misuse.
Qian 2024	Wrong population	The study only mentions patients with overuse disorder, but it is not stated whether this is due to prescription drug misuse.
Rioux 2023	Wrong study	It is a cost-benefit analysis
Tse 2022	Wrong population	Injectable OAT (iOAT) typically involves self-administration of short-acting opioids. It is not clear whether they are participants with prescription drug misuse.
Velez 2021	Wrong objective	The analysis evaluated the net impact of reSET-O on medical costs among actively engaged reSET-O patients using real-world observations.
Barocas 2022	Wrong population	The study only mentions patients with overuse disorder, but it is not stated whether this is due to prescription drug misuse.

Fairley 2021	Wrong population	The study only mentions patients with overuse disorder, but it is not stated whether this is due to prescription drug misuse.
Jawa 2023	Wrong population	The study only mentions patients with overuse disorder, but it is not stated whether this is due to prescription drug misuse.
Scheidell 2024	Wrong population	The study only mentions patients with overuse disorder, but it is not stated whether this is due to prescription drug misuse.
Townsend 2020	Wrong population	The study only mentions patients with overuse disorder, but it is not stated whether this is due to prescription drug misuse.
